# Long-Term Survival After Curative Resection of a Primary Clear Cell Carcinoma of the Pancreas: A Report of a Case

**DOI:** 10.7759/cureus.73357

**Published:** 2024-11-09

**Authors:** Helen Bolanaki, Christina Nikolaou, George Pappas Gogos, Anastasios J Karayiannakis

**Affiliations:** 1 Second Department of General Surgery, Democritus University of Thrace, Medical School, Alexandroupolis, GRC; 2 Second Department of Surgery, Democritus University of Thrace/University Hospital of Alexandroupolis, Alexandroupolis, GRC; 3 Second Department of Surgery, Democritus University of Thrace, Medical School, Alexandroupolis, GRC

**Keywords:** clear cell, clear cell cancer, long term survival, neoplasm, pancreas, primary, resection, surgery, survival

## Abstract

Clear cell pancreatic carcinoma, as a primary lesion, represents a rare malignant entity with very few references. As for the incidence, the clinical characteristics and the prognosis are still to be defined.

Here, we present the case of a 73-year-old female who presented with epigastric pain radiating to the back, anorexia, and dyspepsia. Abdominal computed tomography (CT) showed a well-circumscribed, low-attenuating tumor with peripheral enhancement, arising from the upper border of the pancreatic head. There were no metastases or other primary tumors. Radical resection of the tumor was undertaken. Histopathology showed round-to-oval neoplastic cells with a well-defined cell membrane, prominent cell borders, abundant clear cytoplasm, and centrally located nuclei. The periodic acid-Schiff reaction was positive and a diagnosis of primary clear cell pancreatic carcinoma was made. No adjuvant treatment was given. She remained under regular follow-ups with abdominal and thoracic CT scans for seven years without evidence of recurrence of other primary tumors. She was deceased 87 months after tumor resection because of conditions unrelated to the disease.

This is the first case to the best of our knowledge, of long-term survival after radical resection of a primary pancreatic clear cell carcinoma, suggesting surgery as a treatment option for this rare tumor and review of the relevant literature.

## Introduction

Clear cell carcinomas arise most frequently in the ovaries, kidneys, adrenals, and the lung. Primary clear cell adenocarcinoma of the pancreas is a rare “miscellaneous” carcinoma according to the WHO classification [1]. These tumors are ill-described and poorly understood whereas the clinical aspects of the disease are obscure because very few cases have been reported in the literature [2-11]. Here, we present a patient with primary clear cell pancreatic adenocarcinoma and long-term survival after radical resection of the tumor and review the relevant literature.

## Case presentation

A 73-year-old woman with a BMI of 24.14 kg/m^2^ presented with a two-month history of epigastric pain radiating to the back, anorexia, and dyspepsia. Her past medical history was unremarkable and she denied smoking or alcohol consumption. Clinical examination revealed mild tenderness over the epigastrium, and no other signs were found. 

Laboratory tests including serum amylase level, liver, and renal function tests were all within normal limits. Serum levels of carcinoembryonic antigen (CEA) and carbohydrate antigen 19-9 (CA 19-9) were elevated at 8.7 ng/mL (normal range, 0.9-5.4 ng/mL) and 129.8 U/mL (normal range, 0-30 U/mL), respectively (Table 1).

**Table 1:** Laboratory tests of the patient Cre, creatinine; SGPT, serum glutamic-pyruvic transaminase; SGOT, serum glutamic-oxaloacetic transaminase; ALP, alkaline phosphatase; γ-GT, gamma-glutamyl transferase; LDH, lactate dehydrogenase; TBIL, total bilirubin; DBIL, direct bilirubin; Alb, albumin; AMY, amylase; PT,  prothrombin time; INR, international normalized ratio; CEA, carcinoembryonic antigen; CA 19-9, carbohydrate antigen 19-9.

Test	Value	Normal range
Urea	42 mg/dL	10-50 mg/dL
Cre	0.8 mg/dL	0.5-1.4 mg/dL
SGOT	32 U/L	<38 U/L
SGPT	37 U/L	<41 U/L
ALP	118 U/L	40-129 U/L
γ-GT	51 U/L	8-51 U/L
LDH	329 U/L	240-480 U/L
TBIL	0.9 mg/dL	<1.1 mg/dL
DBIL	0.2 mg/dL	<0.35 mg/dL
ALB	3.8 mg/dL	3.4-5.0 mg/dL
Ferritin	102.34 mg/mL	30-400 mg/mL
AMY	86 U/mL	60-120 U/mL
PT	11 s	9.5-12 s
INR	0.8	0.9-1.1
CEA	8.7 ng/mL	0.9-5.4 ng/mL
CA 19-9	129.8 U/mL	0-30 U/mL

**Figure 1:**
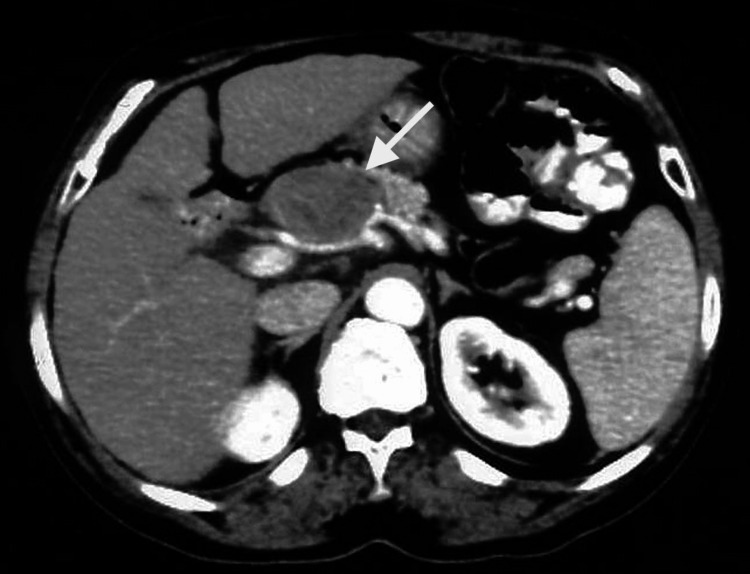
A well-demarcated, round-shaped, low-attenuated mass in the pancreatic head with peripheral enhancement (arrow)

Contrast-enhanced computed tomography (CT) of the abdomen revealed a large (5x4 cm), well-circumscribed, low-attenuating tumor with peripheral enhancement, arising from the upper border of the pancreatic head and extending between the hepatic and splenic artery (Figure 1). 

There were no other lesions, enlarged regional lymph nodes, or liver metastases. A thoracic CT was normal. 

**Figure 2:**
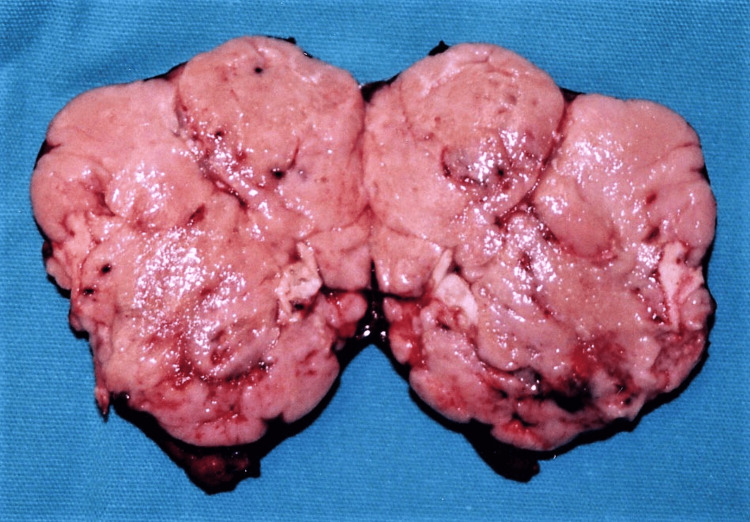
Gross appearance and cut surface of the tumor

At operation, a soft, round-shaped, well-demarcated tumor was found arising from the upper edge of the pancreatic head with an exophytic growth and connected to the pancreas by a thin bridge of apparently normal pancreatic parenchyma. The tumor was encapsulated by a fibrous capsule and extended into the retroperitoneum and upwards between the proper hepatic and splenic arteries. There was no invasion to the adjacent tissues or lymph node involvement. The tumor was dissected free from the retroperitoneum and the celiac trunk and removed en-block with a rim of pancreatic head parenchyma. Regional lymph nodes around the celiac trunk were dissected for histology. The resected tumor measured 4.5x3.5 cm. The cut surface was whitish pink without necrotic or hemorrhagic areas and fibrous septa (Figure 2). 

**Figure 3:**
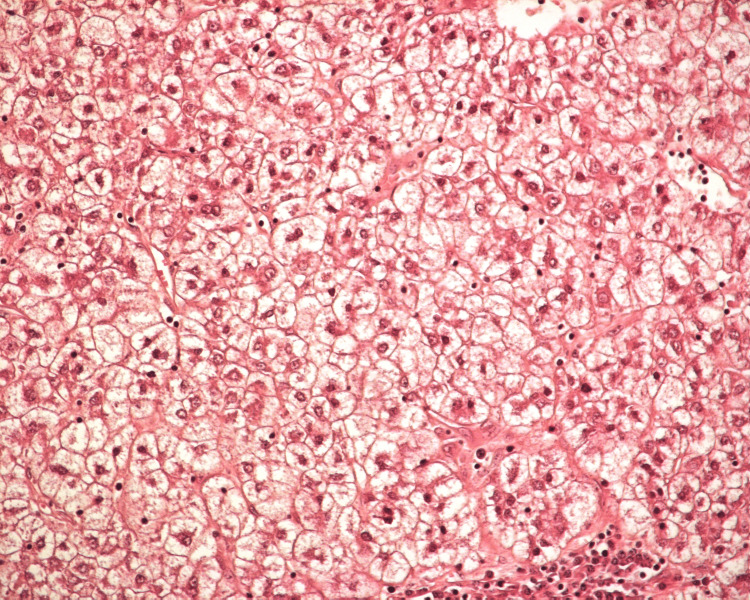
Microscopically the tumor was composed of round-to-oval cells with small centrally located nuclei, well-defined cell membranes, and abundant clear cytoplasm (H&E×200)

Microscopically, the tumor was composed exclusively of clear cells arranged on solid surfaces with scanty fibrous stroma (Figure 3). 

The neoplastic cells were round to oval, with well-defined cell membrane and prominent cell borders, abundant clear cytoplasm, and small centrally located nuclei. The periodic acid-Schiff reaction was positive. 

The patient’s postoperative course was uneventful and she was discharged on the eighth postoperative day. No adjuvant treatment was given and she was scheduled for regular follow-ups with abdominal and thoracic CT scans. She remained well and disease-free for seven years without evidence of recurrence. Ovarian, renal, or lung lesions were not detected during this period. She deceased 87 months after tumor resection because of pulmonary embolism developed after a hip fracture operation.

## Discussion

Clear cell carcinomas are relatively common tumors of the kidneys, female genital tract, adrenals, and the lung. Primary clear cell adenocarcinoma of the pancreas was initially described by Cubilla and Fitzgerald on autopsy in 1980 [1]. Subsequently, very few cases have been reported in the literature [2-15]. Nevertheless, it remains an ill-described and poorly understood entity without detailed information regarding the histopathological features of the tumor and the clinical aspects of the disease. The diagnosis relies mostly on the presence of characteristic neoplastic cells with abundant clear cytoplasm, prominent cell boundaries, and centrally located or eccentric round nuclei without prominent nucleoli occupying most of the tumor, as was the case in our patient [4-6]. Immunohistochemistry, hepatocyte nuclear factor 1 beta (HNF1B) expression, and k-ras mutational analysis are complementary methods to differentiate primary pancreatic clear cell carcinoma from other pancreatic tumors, conventional ductal adenocarcinoma, and metastatic clear cell carcinomas of other locations [14-16].

Several pancreatic lesions such as neuroendocrine tumors, solid serous cystadenoma, solid pseudopapillary tumor, clear cell “sugar” tumor (PEComa), ductal adenocarcinoma with a clear cell component, mixed ductal-endocrine carcinoma, and anaplastic carcinoma may show focal clear cell features and they should be excluded. The occurrence and extent of clear cell morphology, growth pattern, and the immunohistochemical and molecular profiles of these lesions are helpful for their differentiation [3,4,16]. Immunohistochemical positivity for neuroendocrine markers (synaptophysin and chromogranin) differentiates a neuroendocrine tumor and a mixed ductal-endocrine carcinoma. The so-called “sugar” tumor (PEComa) has a solid growth pattern and an epithelioid appearance but does not express epithelial markers and show immunohistochemical melanoma-associated antigen HMB-45 (human melanoma black) positivity [17]. The expression of epithelial markers (CK7, CK20, and CAM5.2) along with CEA positivity and k-ras oncogene mutations suggest a ductal phenotype, whereas a solid/cystic pseudopapillary growth pattern with an epithelioid appearance and alpha-1-antitrypsin production is more in keeping with solid pseudopapillary tumor [4-6,17,18]. Metastatic clear cell carcinoma from other locations notably renal cell carcinoma should be also ruled out [19,20]. In our patient, imaging studies failed to detect any extra-pancreatic primary tumors, either preoperatively or during the long-term follow-up, thus providing confidence that this was a case of true primary pancreatic clear cell carcinoma.

Because of the rarity of this tumor and the small number of reports in the literature, the incidence, demographics, clinical features, proper treatment, and prognosis have not been fully characterized. Based on the present case and previously published reports, it is evident that the tumor most commonly involves the pancreatic head (seven cases), followed by the body (five cases) and tail (three), and affects more frequently men (nine males and six females). The age of the patients ranged between 46 and 75 years (mean age 64.66 years, median age 65 years). The clinical symptoms are nonspecific with upper abdominal pain, loss of appetite, and weight loss being the most common presenting symptoms. Jaundice may be present in cases of multiple liver metastases or when the tumor is located in the pancreatic head causing biliary obstruction. Tumor markers CEA and CA 19-9 are usually within normal limits or elevated mainly when there is disseminated disease with liver metastases (Table 2).

**Table 2:** Clinical features of previously reported cases of pancreatic clear cell carcinomas N/A, not available; CEA, carcinoembryonic antigen; CA 19-9, carbohydrate antigen 19-9.

Age/Sex	Location	Symptoms	Tumor markers	Treatment	Survival	Ref.
57/M	Body, extensive metastases	Abdominal pain, jaundice, ascites	N/A	None	Died six weeks after diagnosis	[2]
71/M	Body and tail, disseminated metastases including the lungs	Epigastralgia	CEA: 628 ng/mL, CA 19-9: 9,900 U/mL	Mitomycin C 5-FU	Died 51 days after diagnosis	[3]
53/M	Head, liver metastases 11 months after diagnosis	Abdominal pain, weight loss, obstructive jaundice	CEA: normal, CA 19-9: 0.3 ng/mL	Partial pancreatectomy	N/A	[4]
75/M	Tail	Right upper quadrant pain	N/A	Distal pancreatectomy	Alive two months after surgery	[5]
61/F	Body	Epigastric pain and weight loss	CEA: 1.1 ng/mL, CA 19-9: 4.4 U/mL	Total pancreatectomy, adjuvant 5-FU	Alive 35 months after surgery	[6]
46/M	Head, omental metastasis	Left hypochondrium pain, anorexia, weight loss	CEA: 2 U/mL, CA 19-9: 2 U/mL	Biopsy of omental lesion	Died three months after surgery	[7]
60/M	Head and body	Epigastric discomfort	N/A	Aspiration biopsy, adjuvant chemotherapy, and radiotherapy	Died four months later	[8]
75/M	Head	Upper abdominal dyspepsia, jaundice, weight loss	N/A	Whipple’s procedure	Died six months after surgery	[9]
66/F	Tail, hepatic metastases	Postprandial epigastric pain, anorexia, weight loss	CEA: 12.17 ng/mL, CA 19-9: 597.1 U/mL	Palliative gemcitabine	Died one month after diagnosis	[10]
74/F	Body and tail, multiple liver metastases	Epigastric pain, anorexia, weight loss	CEA: >600 U/mL, CA 19‑9: >7,000 U/mL	Liver biopsy	N/A	[11]
64/M	Distal body and tail	Epigastric pain, weight loss	CEA: 18.56 ng/mL, CA 19-9: 649.15 U/mL	Distal pancreatectomy	Liver metastasis three months after surgery, received gemcitabine chemotherapy, died two months later	[12]
60/F	Head	Epigastric pain, weight loss	CEA: 2.3 ng/mL, CA 19-9: 170 U/mL	Neoadjuvant FOLFIRINOX, radiotherapy, Whipple’s procedure		[13]
63/F	Pancreatic neck, multiple liver metastases	Upper abdominal pain, dyspepsia, weight loss	CEA: 4 ug/L, CA 19-9: 916 916 kU/L	Liver biopsy, gemcitabine nab-paclitaxel	Alive after two months	[14]
72/M	Body and tail	Incidental findings	CEA: 2.3 ng/mL, CA 19-9: 121 U/mL	Laparoscopic pancreato-splenectomy, postoperative radiotherapy, two months later local recurrence adjuvant FOLFIRINOX administered 12 times	Alive 11 months after surgery	[15]
70/F	Head	Epigastric pain, anorexia, dyspepsia	CEA: 8.7 ng/mL, CA 19-9: 129.8 U/mL	Radical resection	87 months	Present case

CT features of the tumor are not specific for accurate diagnosis but CT scans are useful in excluding other extra-pancreatic primary tumors or metastatic disease [20]. In most reported cases, the tumor was metastatic or unresectable at the time of diagnosis with an aggressive behavior and poor outcome. Multidisciplinary treatments including surgical resection, radiation therapy, and chemotherapy may prolong survival but the overall prognosis is dismal. Even after radical resection of the tumor in cases of localized disease, the prognosis was very poor. Nevertheless, radical resection is the only option in the absence of effective chemotherapy. This is the first case of primary pancreatic clear cell carcinoma amenable to radical resection and with long-term survival.

## Conclusions

Primary clear cell adenocarcinoma of the pancreas is a barely documented and rare malignancy. Information regarding the incidence, treatment modalities, and prognosis of this entity is limited in the literature. Here we presented the first case of long-term survival after radical resection of a primary pancreatic clear cell carcinoma suggesting surgery as a treatment option for this rare tumor. Nevertheless, more cases are needed to clarify the nature of the disease and to elucidate the appropriate therapeutic approach, especially in cases of recurrence or metastatic disease.
